# Tuberculin in Practice

**Published:** 1894-01

**Authors:** Cooper Curtice

**Affiliations:** Moravia, N. Y.


### TUBERCULIN IN PRACTICE.

By Cooper Curtice.

Should there be no other benefit derived from the use of tuberculin than the detection of tuberculous cows, humanity will still have derived inestimable benefit, from sanitary, scientific and economic standpoints.

So much has been published regarding the merit of this diagnostic agent to establish its use that the writer accepts the merit as an assured fact and enters into a rambling discussion of a part of his experience with it, more from the position of one who is endeavoring to eliminate the errors arising from its use rather than one assailing its virtues.

In suggesting that errors may arise from its use I wish first of all to assert that many a seeming error may not be an error at all. For instance: if an animal has a reaction over 106 degrees F. in a tuberculous herd and no cheesy tumors or tuberculous lesion is found on post mortem examination, I for one am going to refrain from pronouncing that animal as not affected when the tuberculin has pointed out and post mortem proven in the same herd that thirteen other head out of sixteen chosen for slaughter were positively tuberculous. If this be the case for a high temperature, at what lower temperature shall we make the dividing line between suspected and not suspected cattle on our post-mortem examinations?

The tuberculin test as used in our New York State Board of

Health work, consists in injecting under the cow's skin from four to eight drops of Tibbertz' *Tuberculium Kochii*, diluted with a half of 1 per cent. carbolic solution to a 10 per cent. solution. At the time of injection, about nine o'clock in the evening, the first temperature is taken. This is done as a matter of convenience; it sometimes enabling us to arrive at a closer conclusion regarding one or another animal. It may be more scientific to take temperatures for the twenty-four hours preceding, but being averse to protracting the expense of examination and the attendant inconveniences to the cattle and dairymen, we have reduced all work to a minimum. So far I am not yet convinced that a longer examination would be more thorough or less prolific of error. On the contrary, I believe the shorter time to be the better. In the following morning, temperatures are taken at intervals of two to three hours, usually two and one-half hours, commencing at seven or eight o'clock, or ten to eleven hours after the injection. This examination is continued to three or four o'clock, or even five, in the afternoon, if the observer chooses. I have found, however, that the earlier time furnishes me the necessary data. After the taking of the second temperature in tuberculous herds, the work is lessened by no longer taking the temperature of those with positive reaction, say 106–108 degrees, for really but one such temperature is necessary to condemn the animal. The afternoon work concerns itself with the uninfected and the incipient cases, continued examination being necessary to ascertain that they have no reactionary fever. So far I find that my diagnoses are more complicated by the late work in the afternoon, and post-mortems show that the morning and early afternoon work is the more reliable.

There are two practical points in examinations that may assist beginners; of course the experienced have their own methods preferable to all others. In injecting, being right-handed, I stand on the right ide of the cow, by her shoulder. By reaching over to the left side I pinch up the skin upon the left shoulder just behind the scapular spine, and thrust the hypodermic needle with syringe attached into the fold, near my thumb, with my right. The syringe is held in the hollow of my hand, the needle between my fingers and thumb. With my elbows and body I get such a purchase on the cow's shoulders that when they move I move with them and so avoid mishap. They may either move toward me, i. e., away from the needle, or "buck." Fortunately, few do the latter. A few have gone to their knees. Usually with sharp needles there is no movement. I make no attempt at disinfection of hides, but use great care with needles, syringe, etc.

In taking anal temperatures I use four thermometers. Five have been tried, but the extra one causes more loss and does not seem to be much help. I find that after waiting two and one-half minutes for the first to register, it requires from two and one-half to three minutes to get through the set. As soon as a temperature is read the thermometer is inserted into the next animal. Needless to say that one has to be absolutely sure that the mercury is shaken down from the last reading or else one must try again. In taking these anal temperatures one also has to be on the watch against the cattle ejecting them on to the floor. This is more liable to occur toward noon or in the afternoon. It is well to have extra thermometers to replace broken ones. In summer work the cattle were permitted to go afield after injecting, keeping them up only during the following day. The cattle are not allowed to water until after the test is through.

The thoroughness with which tuberculous cattle may be selected from a given herd depends on the tuberculin, on the cattle and their conflicting conditions, and on the inspector.

We presuppose that two of these factors, the tuberculin and the inspector, are known and constant; that the presence or absence of tuberculosis is the unknown quantity. There is some reason for supposing, however, that in the course of our tests with various bottles of tuberculin there is a difference in intensity of action even in the Tibbertz' material. For instance, I had supposed until lately that reaction of 102.7 or 102.8 degrees F. indicated a tuberculous patient in herds with marked cases. This was pointed out by marked success in picking out the well cattle or cattle with no reaction in a number of herds, as proven by slaughter of all. Lately, however, I have tried herds and got low fever temperatures to above 104 degrees without any tuberculous lesions being found. This indicates that for some reason there is a reaction produced by tuberculin different from that in earlier herds.

The dose of 0.25 cubic centimetres is the one oftenest used by us. 0.3 c. c. have been used with, I believe, more pronounced results. Larger doses are now being tried.

In the succeeding paragraphs it will be understood that for our purposes the intensity of the tuberculin is regarded as a constant quantity, and the unknown differences supposed to exist in the cow. Tuberculin seems to fail often in some of the worst cases, or cases which one can diagnose as tuberculous by physical inspection. Cow 87, whose temperature ranged between 102 degrees before injection and 108.1 degrees the morning after, was one of the bad ones in the herd. Cow 216, 102 degrees evening temperature and 100.8 degrees highest morning temperature, was the worst in her herd. Cow 261, evening temperature 104.2 degrees, highest morning 104.8 degrees, was so bad that she could not be driven from the farm to place of slaughter of the herd. Cows 306, evening temperature 103.8 degrees, highest morning temperature of 102 degrees, and No. 311, 103.2 degrees evening, 103.6 degrees morning, were typical wasters and turned aside to die. A larger quantity of tuberculin might have been necessary to create the required fever in these animals, but how shall we judge of the quantity required to give each? If we know they are tuberculous why do we try them, and if not why should we vary the amount given?

The highest temperature that I find is one of 108 degrees, in 348, a badly diseased two-year-old registered Guernsey bull. In this the external glands and the testicles were tuberculous, carrying nodes an inch across. Cattle with the higher temperatures appear to be in all stages of the disease.

The fine work of the inspector is demanded in judging of the low temperature; those ranging from 102.5 deg. to 105 deg., as previously stated. I have until lately supposed that reactions between these might be indicative of disease, and they may have been, but recent herds tried and selected on the same basis indicate slight reaction from other causes.

Cows 643, 104.4 deg.; 644, 105.4 deg.; 645, 107.6 deg.; 658, 106.2 deg.; and 655, 102.8 deg., were the only tuberculous animals, (as determined by the finding of cheesy tumors) out of seventeen selected. Other temperatures ranged between 105 deg. and 102.7 deg.

Cows 667, 107.2 deg.; 675, 107.8 deg.; 677, 107.4 deg.; and 670, 102.4 deg. were the only tuberculous animals out of thirteen head selected. Other temperatures varied between 102.4 deg. and 103.8 deg.

On the contrary, in five other herds—Adams, Dart, Birdsall Scott and Shaw—any temperatures above 102.5 deg. or 102.7 deg. belonged to a tuberculous animal, and the only apparently sound cattle had lower temperature.

Frequently tuberculous animals, with but little disease and indicating little or no reaction with tuberculin, have come to notice, thus: 655, 102.8 deg.; 670, 102.4 deg.; 52, 102.4 deg.; 58, 102.6 deg.; 253, 101.7 deg.; 242, 102.1 deg., and others.

Cases of considerable rise, without the tubercles being found on post-mortem examination, are altogether too frequent, thus: 673, 106.4 deg.; 648, 105 deg.; 571, 106.1 deg.; No. —, 104.6 deg. and others.

Other diseases, as abscesses and bronchitis, complicate matters, but do not begin to enter into the diagnosis like high temperature in apparently sound cattle. Bronchitis and pneumonia of scattered lobes seem to create no markedly high rise in temperature. Even advanced cases of broncho-pneumonia rarely give any reaction.

After the character and intensity of the tuberculin, the most important element to be taken into consideration is the presence of a fœtus in the last stages of gestation. In heifers and young cows the attendant fever or the fever obtained the next day after injection is frequently two or even three degrees. To eliminate this source of error one has to learn, as nearly as possible, the age of the fœtus, and base judgments upon it.

Hitherto when deciding upon the presence of tuberculous cattle in a herd, if there were no temperatures over 103.5 deg. or 104 deg., and there was no history of tuberculosis as determined by the previous killing or death of a cow, I have felt quite safe in passing them as free. If, however, high reactions indicated the presence of the disease in some, I have felt justified in taking cattle with quite low temperatures, and until recently have not been disappointed. In but few instances have I been assured that all the tuberculous animals of a herd had been selected and in those the herd was condemned. These herds have fully demonstrated, however, that not more than a single incipient would be left. At present the only way apparent to rout out the disease seems to be to kill those known to be tuberculous, quarantine others and retest at some remote date. The quarantine need extend over the cattle only in so far as exchange is concerned, the butter and milk being allowed as before. If the owner decided to beef one or more it should be his privilege, as the menace from that one at least would be removed. The proof of slaughter should be exacted.

The problems of selecting tuberculous animals by the use of tuberculin and of selecting all the tuberculous animals of a herd, are vastly different in execution. The first can be done almost ninety-nine times out of a hundred. The latter may involve many innocent animals. With the perfection of tuberculin itself, of the methods in using it and of our skill in making deductions from the facts, we shall be able to clean a herd of this disease in the majority of trials, and this act is of such importance to public hygiene and the dairy industry that it easily places tuberculin among the foremost of specifics.

As yet it seems to me that there are no data upon which the actual percentage of tuberculous cattle to the whole number can be based.

The percentage of tuberculous animals in a given herd may be high, anywhere from 30 per cent. to 100 per cent., but tuberculous herds do not form a large proportion of our dairies. The percentum found to obtain in the slaughter of cows in public abattoirs, viz., of 2 per cent. to 3 per cent., seems to me to be a very fair estimate. Herd to herd inspection of last winter, by physical diagnosis, yielded to me ½ of 1 per cent. The slaughter of an entire herd on the supposition that most cattle in it were diseased, raised this percentage, but not above the previous estimate.

With tuberculin and plenty of inspectors, it is going to be a serious task to eradicate the 2 per cent. tuberculous cattle from the herds of New York; but it is feasible and promises the best of results, and certainty of fulfilment. Without tuberculin scarcely the badly diseased, or those selected for death in the near future, can be chosen.

To prosecute hygienic work with tuberculous cattle successfully, we must use tuberculin; especially on herds in which tuberculosis is suspected or known to exist. Until the majority of these are cleaned up, I can not counsel its use, save, perhaps, in weeding herds whose owners must guarantee the offspring and others as free of this disease. Until we are surer of our deduction regarding reactions, had we not better handle their herds with great caution, enforcing quarantine until a future test shall have decided upon disease in suspects, rather than indiscriminately slaughter them? Quarantine of suspects need involve no expense or loss other than that entailed by loss of immediate sale, of individual cattle, the owner being permitted in all other ways to use the products as before, and not, unless he prefers, being required to separate them from others.

If this article serves to point out wherein we may err in the use of tuberculin, and adds proof of faith in its utility in State inspection, its purpose will be fulfilled.

Moravia, N. Y.

